# Dense, uniform, smooth SiO_2_/TiO_2_ hard coatings derived from a single precursor source of tetra-*n*-butyl titanate modified perhydropolysilazane

**DOI:** 10.1039/c8ra02289e

**Published:** 2018-05-08

**Authors:** Zongbo Zhang, Dan Wang, Fengyan Xiao, Qianying Liang, Yongming Luo, Caihong Xu

**Affiliations:** Research/Education Centre for Excellence in Molecular Sciences, Institute of Chemistry, Chinese Academy of Sciences Beijing 100190 China zongbo@iccas.ac.cn luoym@iccas.ac.cn caihong@iccas.ac.cn; School of Chemistry and Chemical Engineering, University of Chinese Academy of Sciences 100049 China

## Abstract

SiO_2_/TiO_2_ composite coatings are of great interest due to their optical, photocatalytic, electrical and mechanical properties. There are many methods for preparing SiO_2_/TiO_2_ composite coatings. Among these, the sol–gel process is eco-friendly and easily implementable for industrial applications. However, it is still a great challenge to prepare dense, uniform and smooth SiO_2_/TiO_2_ composite coatings with a sol–gel process. In this work, we show a modified sol–gel process for achieving dense, uniform and smooth SiO_2_/TiO_2_ coatings with tetra-*n*-butyl titanate modified perhydropolysilazane (PHPS) as a single precursor source, in which PHPS acts as the source for SiO_2_ instead of a traditional alkoxylsilane compound. The micro-morphology and composition evolution during the preparation process have been investigated; a smooth surface with a roughness average (*S*_a_) below 4.5 nm and a uniform distribution of Ti elements over the entire coating are shown. These dense, uniform SiO_2_/TiO_2_ coatings exhibit excellent mechanical robustness, with hardness values as high as 9.45 GPa, excellent optical transparency and hydrophilic properties.

## Abstract 2

Dense, uniform, smooth SiO_2_/TiO_2_ hard coatings derived from a single precursor source of tetra *n*-butyl titanate modified perhydropolysilazane.

## Graphical abstract

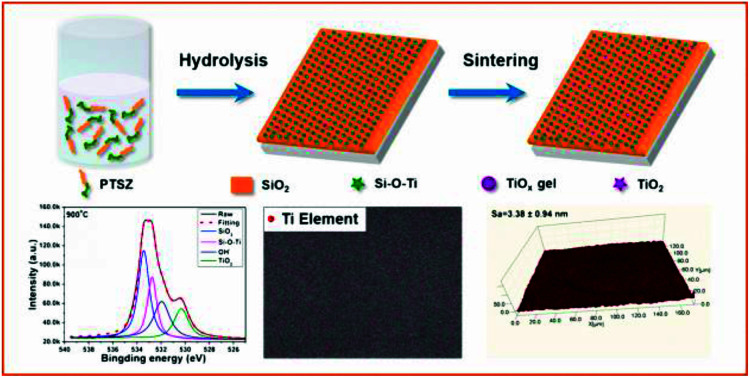

## 1. Introduction

SiO_2_/TiO_2_ coatings and their multilayer packing have being widely investigated and applied in the fields of optical waveguiding, antireflection coatings, thermal protection systems, self-cleaning coatings, and semiconductor devices, due to their excellent optical, photocatalytic, superhydrophilic, electrical, and mechanical properties.^1–8^ Therefore, various methods have been developed to prepare SiO_2_/TiO_2_ coatings, such as electron-beam evaporation, chemical vapor deposition (CVD), and the sol–gel process. Among these, the sol–gel method is simple and inexpensive, which makes it especially suitable for the preparation of coatings on components with complex surfaces or large dimensions. Thus, there are numerous reports on the preparation of SiO_2_/TiO_2_ coatings using the sol–gel method.

Generally, alkoxylsilane and tetra-alkyl orthotitanate are the main raw materials for the sol–gel route, which involves hydrolysis, condensation, and sintering processes. Due to the faster hydrolysis rate of Ti precursors, the co-hydrolysis of alkoxylsilane and tetra-alkyl orthotitanate usually leads to an inhomogeneous dispersion of SiO_2_ and TiO_2_ sols, which results in aggregates, cavities, and rough surfaces in the formed SiO_2_/TiO_2_ coating.^9,10^ Actually, the inhomogeneous status will seriously affect the optical properties and mechanical robustness of the achieved coatings.^4^ Thus, some efforts have been made to solve this problem, such as carrying out pre-hydrolysis of the Si precursor,^11^ adopting organic stabilizers to control the hydrolysis,^9,12^ altering the alkoxyl groups in the Ti and Si precursors,^10,13^ and using a non-hydrolytic sol–gel process.^14^ Unfortunately, these methods still produce SiO_2_/TiO_2_ coatings with high roughness. Recently, HS Chen reported that the co-hydrolysis-condensation of acac-stabilized Ti and an activated Si precursor achieved a homogeneous SiO_2_/TiO_2_ coating with a uniform dispersion of Ti atoms in all directions of the coating.^10^ Apparently, complicated steps must be undertaken in advance to ensure efficient preparation. Therefore, it is still challenging to prepare uniform SiO_2_/TiO_2_ coatings using a more convenient method.

Recently, perhydropolysilazane (PHPS) has drawn more and more attention as a precursor for dense, uniform and scratch-resistant SiO_2_ coatings. By exposing as-deposited PHPS coatings to high temperatures, moistening atmospheres, aqueous ammonia vapor, hydrogen peroxide solutions, or deep vacuum ultraviolet (DUV) irradiation at room temperature, SiO_2_ coatings can be easily prepared that exhibit good adhesion to different substrates, and excellent thermal and mechanical stability, as well as high optical quality.^15–23^ In addition, the Si–H and N–H groups in PHPS are highly reactive toward hydroxyl, alkoxyl, and epoxy groups, and halogen ions, which provides the possibility to modify PHPS with other reactive compounds.^24,25^ Based on this, novel types of silicon-based multicomponent ceramics or organic-silica nanocomposites endowed with new properties have been developed.^26–33^ Moreover, the Si–N bonds in PHPS are easily hydrolysed to form Si–OH,^18^ which implies that when using PHPS as a source of SiO_2_ in the preparation of SiO_2_/TiO_2_ coatings, the trickiest problem existing in the traditional sol–gel route may be avoided: namely the slower hydrolysis reaction rate of alkoxylsilane as a source of SiO_2_ compared to the Ti precursor for TiO_2_. However, to our knowledge, there is still no report on the preparation of SiO_2_/TiO_2_ coatings where PHPS is adopted as the source of SiO_2_.

In this work, SiO_2_/TiO_2_ coatings have been successfully fabricated from a single source polymeric precursor, polytitanosilazane (PTSZ), which is synthesized *via* modifying PHPS with tetra *n*-butyl titanate (TBT) at room temperature. The obtained coating is dense and smooth, and the distribution of all elements is uniform. Besides, due to the homogeneity and densification, the coating exhibits excellent optical transparency and mechanical properties. The synthesis of PTSZ has been described and the correlation between the coating properties and the fabrication process has been investigated. This work provides a novel and easily implemented method to prepare dense, uniform TiO_2_/SiO_2_ coatings.

## 2. Experimental

### 2.1 Materials

Liquid PHPS was synthesized *via* the ammonolysis of dichlorosilane (H_2_SiCl_2_) according to the literature.^27^ TBT was purchased from Sinopharm Chemical Reagent Co., Ltd (Beijing, China) and used as received. Xylene was purchased from Beijing Chemical Factory (Beijing, China) and distilled from calcium hydride prior to use. Si (100) and Si (111) single-crystal wafers with an area of 10 mm × 10 mm were purchased from Shanghai Boshuo Sealing Technology Co., Ltd (Shanghai, China). Quartz glass with dimensions of 30 mm × 10 mm × 1 mm was purchased from Jinzhou east quartz material Co., Ltd (Liaoning, China). Prior to coating, the Si (100), Si (111) single-crystal and silica glass substrates were cleaned ultrasonically in acetone, ethanol, and distilled water in sequence, and then dried under nitrogen.

### 2.2 Synthesis of PTSZ

TBT-modified PHPS (PTSZ) was prepared *via* the following procedure. A solution of PHPS in CH_2_Cl_2_ with a concentration of 50 wt% was vacuum-suction filtrated to remove the solvent. Then, liquid PHPS (1 g) and 21 mL of dry xylene were injected into a 50 mL two-necked flask under flowing nitrogen. After PHPS dissolved in the xylene to form a homogeneous solution, TBT (1 g) was added dropwise into the solution. Then the mixture was stirred under a nitrogen atmosphere at room temperature for 24 h to yield a light yellow solution of PTSZ. The obtained PTSZ solution with a 10% concentration was directly used to prepare the SiO_2_/TiO_2_ coatings.

### 2.3 Preparation of SiO_2_/TiO_2_ coatings

**Fig. 1:**
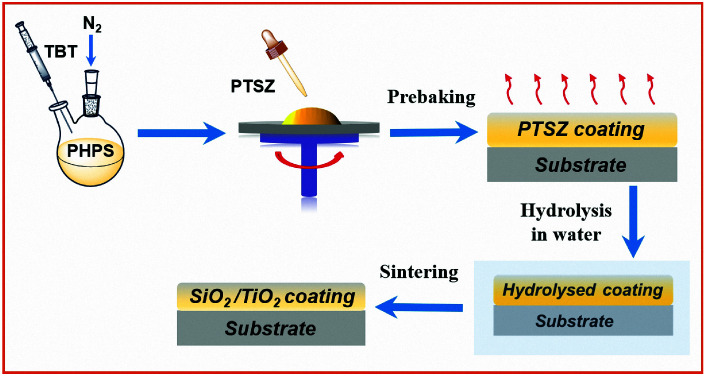
A schematic illustration of the SiO_2_/TiO_2_ coating preparation.

PTSZ solution was coated on the pre-treated Si single-crystal and quartz glass substrates *via* spin-coating at 2000 rpm. The coated substrates were prebaked in an oven at 100 °C for 1 h to remove the solvent (prebaked film) and then hydrolysed in distilled water for 4 h (hydrolysed film). Finally, the hydrolysed samples were annealed at different temperatures in the range of 100 to 900 °C for 1 h under flowing air (Fig. 1).

### 2.4 Characterization

^1^H-NMR spectra were recorded in CDCl_3_ solution using a Bruker AVANCE 400 spectrometer. Fourier transform infrared (FT-IR) spectra of the coatings deposited on Si (100) substrates were recorded using a Bruker Tensor-27 FT-IR spectrometer over the wavenumber range of 400–4000 cm^−1^, using bare Si (100) as a reference. X-ray photoelectron spectroscopy (XPS) data was obtained using an ESCALab220i-XL electron spectrometer from VG Scientific (East Grinstead, UK) using 300 W Al-Kα radiation. X-ray diffraction (XRD) measurements were carried out using a Rigaku D/M4X 2500 diffractometer with Cu-Kα radiation. The surface morphologies and titanium element mapping distributions of the coatings were studied using a Hitachi S-4800 scanning electron microscope (SEM) equipped with an energy dispersive spectroscopy (EDS) system. The surface roughness data for the coatings was obtained using a SOHIO white-light interfering profilometer. The transmittance spectra of the coatings on quartz glass substrates were recorded using a Hitachi U-3210 UV-VIS spectrophotometer over the wavelength range of 300–800 nm. The refractive indices and thicknesses of the coatings deposited on Si (111) substrates were measured using a Sentech-SE800 ellipsometer. The surface hardness and elastic modulus values of the coatings were measured using an MTS XP nano-indenter with a Berkovich diamond indenter. The reported data are average values of five measurements performed on different areas of the samples, with a contact depth ranging from 50 to 100 nm. Considering the fact that the surface hardness and elastic modulus of a coating are easily affected by the substrate, the displacement depth is less than 20% of the thickness of the coating. Coatings with a final thickness of over 1.2 μm, which were prepared by repeating the process of spin-coating and annealing eight times, were used for measurements. The hydrophilic properties of the coatings were evaluated by examining the water contact angle using a DSA100 contact angle meter with a droplet volume of 2 μL.

## 3. Results and discussion

### 3.1 Chemical structure of PTSZ

**Fig. 2:**
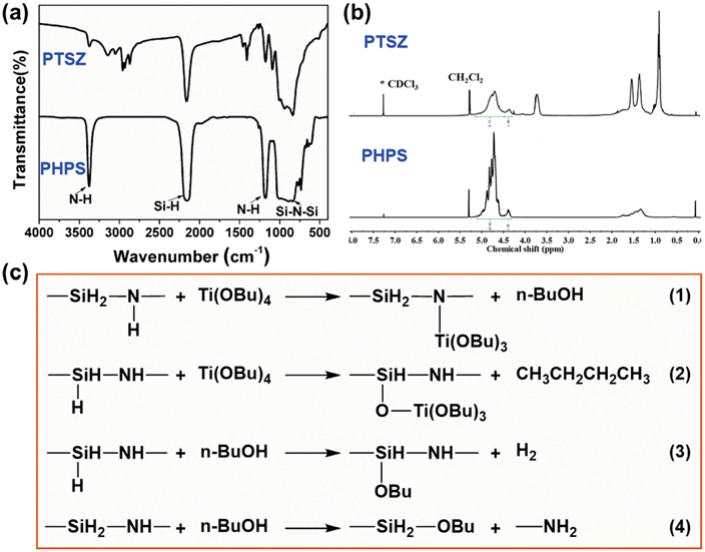
The chemical structures of PHPS and PTSZ: (a) FT-IR spectra; and (b) ^1^H-NMR spectra. (c) Possible reactions between PHPS and TBT.

FT-IR spectra of PHPS and PTSZ are shown in Fig. 2. For PHPS, the absorption at 2160 cm^−1^ can be assigned to Si–H vibrations, and the absorption bands at 3400 cm^−1^ and 1180 cm^−1^ indicate the presence of N–H groups.^24,25,34,35^ Meanwhile, the strong broad bands at 820–1020 cm^−1^ are assigned to Si–N–Si vibrations. In the FT-IR spectra of PTSZ, the intensities of the N–H and Si–H absorption peaks obviously decrease, and stretching vibrations of CH_3_ and CH_2_ groups at 2960 cm^−1^ and 2910 cm^−1^, deformation vibrations of CH_3_ groups at 1400 cm^−1^, and absorption by a Si–O group at 1066 cm^−1^ have been observed. Si–O–Ti or Si–N–Ti bonds form *via* reactions between PHPS and TBT, as shown in eqn (1)–(4)^36,37^ in Fig. 2(c). However, both the absorptions of Si–N–Ti at 1030 cm^−1^ and Si–O–Ti at 935 cm^−1^, formed during reactions between PHPS and TBT, are overlapped by the strong Si–N–Si absorption band at 820–1020 cm^−1^. Thus, it is difficult to separate bands attributed to Si–N–Ti and Si–O–Ti bonds from that of the Si–N–Si bond.

In the ^1^H-NMR spectra of PHPS (Fig. 2(b)), the peak at 4.80 ppm is attributed to SiH/SiH_2_ groups, while the peak at 4.34 ppm is assigned to SiH_3_ groups, and the broad peak at 1.8–1.2 ppm belongs to NH groups.^36^ The peak at 5.51 ppm corresponds to residual CH_2_Cl_2_ in PHPS. Compared to the spectrum of PHPS, the ^1^H-NMR spectrum of PTSZ exhibits new peaks at 4.28 ppm and 3.7 ppm, which are assigned to CH_2_O groups. Meanwhile, CH_2_ and CH_3_ peaks in PTSZ have also been observed, although these are overlapped by the peaks from NH groups between 2.0 and 0.5 ppm. All these data from FT-IR and NMR studies imply that reactions happened between PHPS and TBT, which also indicates that a single polymeric precursor containing Si–N and Si–O–Ti structures has been obtained.

### 3.2 Chemical composition of PTSZ based coatings

**Fig. 3:**
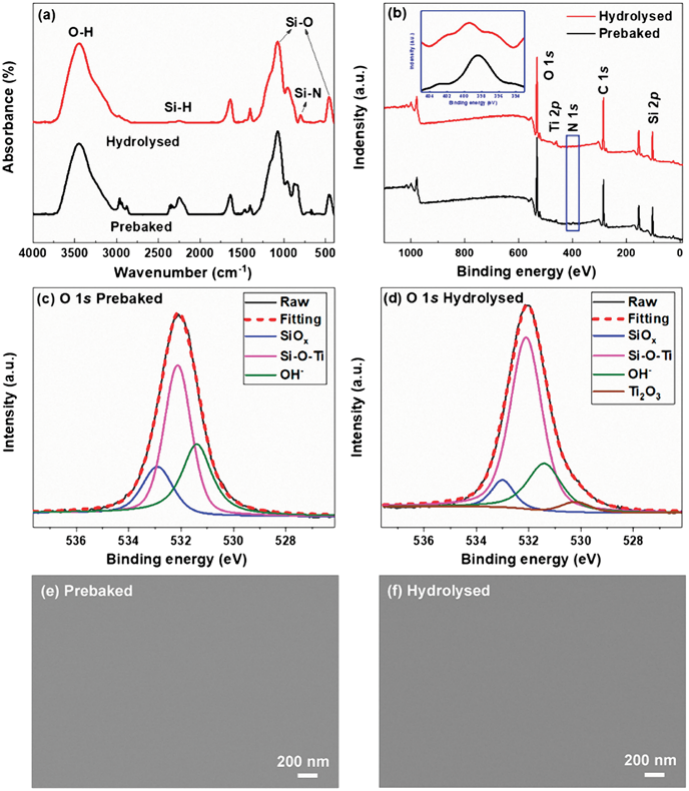
(a) FT-IR spectra and (b) XPS surveys of prebaked and hydrolysed coatings. O 1s XPS spectra of the (c) prebaked coating, and (d) hydrolysed coating. Surface morphologies of the (e) prebaked coating, and (f) hydrolysed coating.

The chemical structure evolution of PTSZ based coatings was studied after the hydrolysis and pyrolysis processes, respectively. As shown in Fig. 3(a), for the hydrolysed coating, the intensities of the absorptions at 2160 cm^−1^, attributed to the Si–H bond, and 820–1020 cm^−1^, assigned to Si–N–Si bonds, show an obvious reduction compared with those of the prebaked PTSZ coating, while the bands assigned to a Si–O–Si asymmetric stretching vibration at 1066 cm^−1^ and a Si–O–Si rocking vibration at 440 cm^−1^ become broader, which can be attributed to hydrolysis reactions of Si–N and Si–H groups in PTSZ, as well as subsequent condensation and cross-linking reactions during the treatment of PTSZ in distilled water. XPS surveys show that the surface of the hydrolysed coating contains unnoticeable N elements. For the prebaked coating, the O 1s XPS spectrum has been deconvoluted to three components, which are assigned to Si–O bonds of SiO_*x*_, centred at 532.9 eV, Si–O–Ti bonds, centred at 532.1 eV, and OH^−^, for the peak at 531.4 eV, respectively (Fig. 3(c)).^38–40^ For the hydrolysed coating, it can be seen that the relative peak area of Si–O–Ti increases from 49.9% to 66.7%, and the Ti–O bonds in Ti_2_O_3_ appear in the peak centred at 530.8 eV. These data suggest that the hydrolysis process has promoted the conversion of PTSZ and formed a relative dense coating, as shown in Fig. 3(e) and (f). It should be mentioned that a reduction in thickness from 352 nm for the prebaked film to 345 nm for the hydrolysed film has been detected, which may also imply that a densification process occurred after the hydrolysis process, by soaking the film in water.

**Fig. 4:**
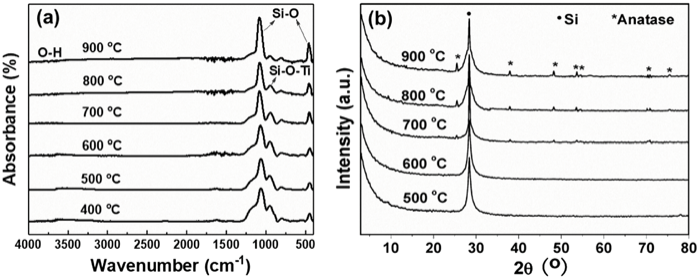
PTSZ based coatings pyrolysed at different temperatures: (a) FT-IR spectra and (b) XRD patterns.

After treatment at 400 °C for 1 h under flowing air, the Si–N–Si group bands disappear, while the absorption by the Si–H groups at 2160 cm^−1^ completely disappears, as shown in Fig. 4(a). With an increase in pyrolytic temperature from 400 to 900 °C, the intensities of the Si–O–Si (1066 and 440 cm^−1^) absorption bands in the PTSZ based coatings gradually increased, while the intensity of Si–O–Ti absorption band at 935 cm^−1^ decreased with increasing temperature, which implies that the pyrolysis process accelerates the conversion of the Si–O–Ti phase to SiO_2_ and TiO_2_ phases above 400 °C.

XRD diffraction patterns of the PTSZ based coatings on Si (111) treated at different pyrolytic temperatures (500–900 °C) under an air atmosphere are shown in Fig. 4(b). There is only one diffraction peak at 28°, deriving from the Si (111) substrate, found in coatings obtained below 700 °C, which indicates the coatings are fully amorphous. After annealing at 700 °C, diffraction peaks at 25.48, 37.98, 48.24, 54.14, 55.28, 70.66, and 75.52°, assigned to the anatase phase of TiO_2_, are observed.^41^ Moreover, the intensities of the diffraction peaks belonging to the anatase phase are enhanced with a rise in pyrolytic temperature, which demonstrates that the crystallization degree of the ceramic coatings has been increased. It should be noted that the diffraction intensities from the anatase phase of TiO_2_ are depressed due to the strong diffraction peak from the Si (111) substrate, which results in the weak TiO_2_ peaks shown in Fig. 4(b).^42^

**Fig. 5:**
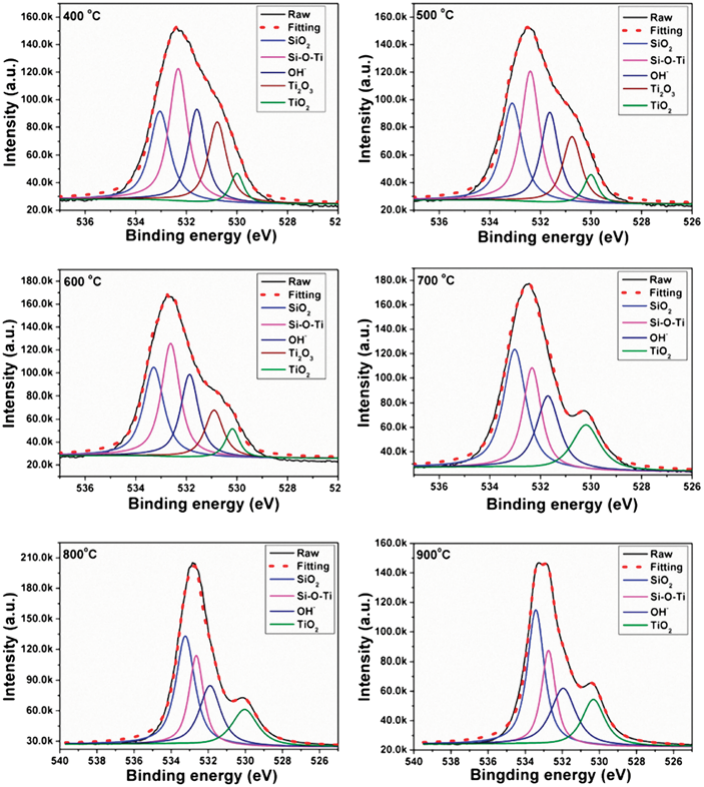
O 1s XPS spectra for PTSZ based coatings pyrolysed at different temperatures.

The compositions of the PTSZ based coatings obtained at different temperatures were further analysed *via* XPS. XPS spectra of the O 1s region are shown in Fig. 5, and the O 1s peak for PTSZ based coatings has been deconvoluted to five components, which are assigned to: Ti–O bonds in Ti_2_O_3_ for the peak centred at 530.8 eV; Ti–O bonds in TiO_2_ for the peak centred at 531.0 eV; OH^−^ for the peak at 531.7 eV; Si–O–Ti for the peak centred at 532.3 eV; and Si–O bonds for the peak centred at 533.2 eV.^2,40,43,44^ As shown in Fig. 5, the peak from Ti–O bonds in Ti_2_O_3_ disappears when the PTSZ based coatings are pyrolysed above 600 °C. The chemical compositions of the coatings, calculated from the deconvoluted O1s spectra, are summarized in Table 1. It can be found that with a rise in pyrolytic temperature, the Si–O–Ti and Ti_2_O_3_ content decreases, while that of SiO_2_ and TiO_2_ increases. This is mainly attributed to the cleavage of the Si–O–Ti bonds, which then transform into SiO_2_ and TiO_2_ phases during the ceramization process. Meanwhile, the Ti_2_O_3_ phase tends to form a TiO_2_ phase at high temperatures.^43^ These data from XPS are consistent with the results from the FT-IR spectra of PTSZ based coatings. The existence of OH^−^ in the SiO_2_/TiO_2_ composite coatings is attributed to H_2_O chemically and physically adsorbed by SiO_2_ and TiO_2_. The content of adsorbed H_2_O increased with an increase in the SiO_2_ and TiO_2_ content in the coatings, and hence resulted in a higher OH^−^ content for coatings obtained at higher temperatures.^40^

**Table 1:** Detailed deconvolution data from XPS peaks

Sample		SiO_2_	Si–O–Ti	OH^−^	Ti_2_O_3_	TiO_2_
400 °C	*E*_b_ (eV)	533.0	532.3	531.6	530.7	530.0
	*r*_i_ (%)^a^	23.02	31.55	20.76	19.40	5.27
500 °C	*E*_b_ (eV)	533.1	532.4	531.6	530.8	530.0
	*r*_i_ (%)	25.90	31.43	20.97	16.32	5.39
600 °C	*E*_b_ (eV)	533.25	532.62	531.87	530.9	530.14
	*r*_i_ (%)	27.03	31.26	22.34	13.24	6.13
700 °C	*E*_b_ (eV)	533	532.33	531.70		530.14
	*r*_i_ (%)	35.32	24.69	22.80		14.81
800 °C	*E*_b_ (eV)	533.25	532.64	531.90		530.12
	*r*_i_ (%)	36.52	23.23	23.05		17.20
900 °C	*E*_b_ (eV)	533.43	532.74	531.92		530.32
	*r*_i_ (%)	36.92	22.03	23.54		17.51

a The ratio *A*_i_/Σ*A*_i_ (*A*_i_ is the area of each deconvoluted peak).

### 3.3 Surface morphologies of PTSZ based coatings

**Fig. 6:**
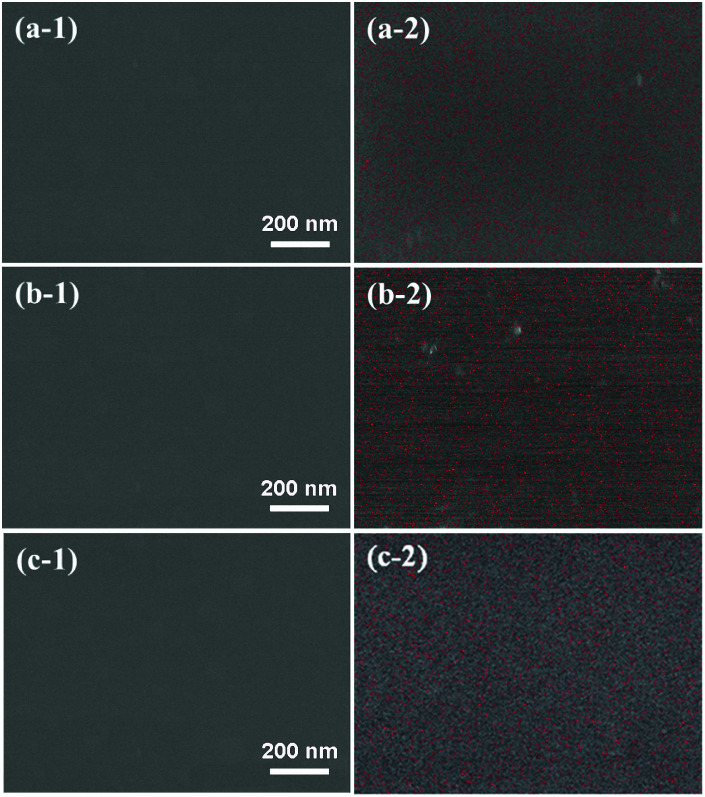
Surface morphologies and EDS maps of PTSZ based coatings treated at: (a) 400 °C; (b) 700 °C; and (c) 900 °C.

**Fig. 7:**
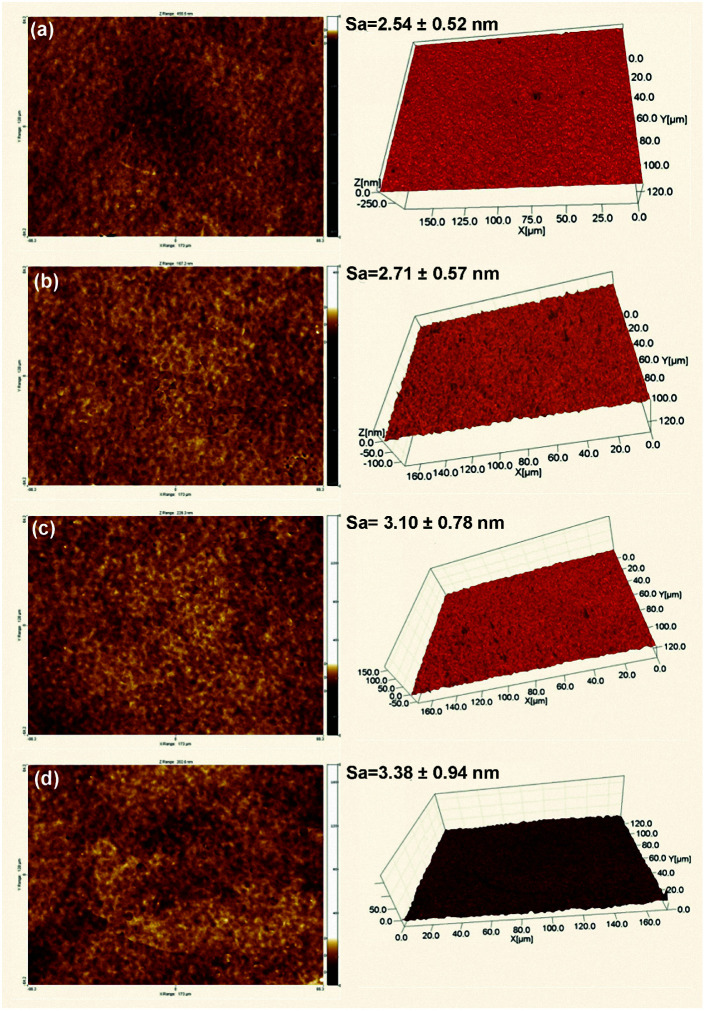
Two- and three-dimensional images of PTSZ based coatings treated at: (a) 400 °C; (b) 600 °C; (c) 700 °C; and (d) 900 °C.

Surface morphologies and EDS maps of PTSZ based coatings on Si (111) treated at different temperatures are presented in Fig. 6. It can be seen that all samples show a uniform, smooth and dense surface without cracks, and the distribution of the element Ti in the coatings is homogeneous. No obvious Ti element enrichment has been observed. Two- and three-dimensional images of PTSZ based coatings annealed at different temperatures are displayed in Fig. 7. It can be clearly seen that all the obtained coatings exhibit smooth morphologies. The surface roughness of the coatings treated at different temperatures is in the scale of several nanometers, which increases with a rise in the annealing temperature. As revealed *via* XRD and XPS, crystalline TiO_2_ was observed above 600 °C, thus destroying the uniformity of the coating. Nevertheless, the roughness is still below 4.5 nm, which is ultra-low and not usually found in previous works involving the traditional sol–gel method. Hence, SiO_2_/TiO_2_ coatings prepared *via* this method are very dense, smooth and homogeneous.

### 3.4 Optical, mechanical and hydrophilic properties of PTSZ based coatings

**Fig. 8:**
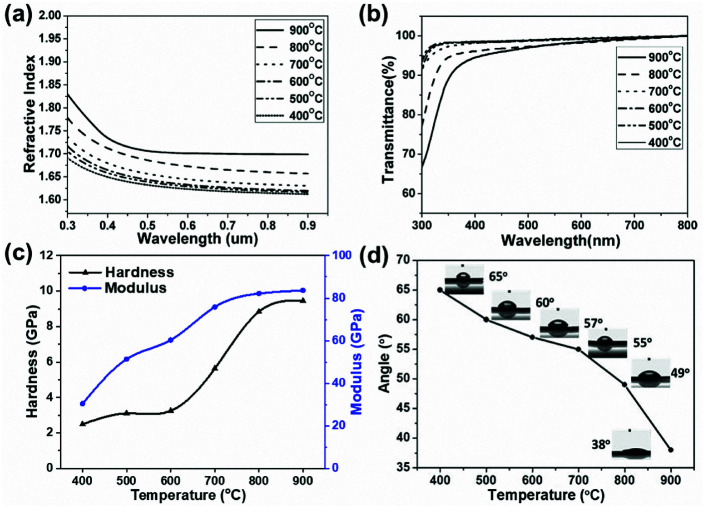
Optical properties of PTSZ based coatings pyrolysed at different temperatures: (a) refractive index; and (b) UV-vis spectra. (c) Mechanical properties of PTSZ based coatings obtained at different temperatures. (d) The water contact angles for PTSZ based coatings obtained at different pyrolytic temperatures.

The refractive indices of the composite coatings prepared at different temperatures (400–900 °C) with respect to wavelength are shown in Fig. 8(a). The refractive index decreases as the wavelength increases from 300 to 900 nm, which is coincident with the phenomenon of dispersion for transparent materials in the visible range. The thicknesses and refractive indices at 630 nm of the coatings, obtained from ellipsometry, are summarized in Table 2. The coating treated at 400 °C has a refractive index of about 1.62, while that of the coating treated at 900 °C is 1.70. The enhancement of the refractive index with increasing temperature is due to the formation of a denser structure for the composite coatings at high temperatures. In addition, with the increase in pyrolytic temperature, more TiO_2_ formed from the cleavage of Si–O–Ti and transformation from Ti_2_O_3_, as discussed in relation to the XPS measurements. The thicknesses of the coatings slightly decrease with an increase in the pyrolytic temperature, due to improved densification at higher temperatures.^10^

**Table 2:** The refractive indices at 630 nm and thicknesses of the coatings

Sample	Thickness (nm)	Refractive index
400 °C	142	1.62
500 °C	131	1.63
600 °C	124	1.63
700 °C	118	1.64
800 °C	106	1.67
900 °C	100	1.70

Fig. 8(b) shows transmittance spectra of the coatings deposited on quartz glass substrates in the wavelength range of 300–800 nm. It can be seen that the transmittance of the coatings prepared at various temperatures is above 90% in the visible region, indicating that the coatings derived from PTSZ are highly transparent. In addition, for the coatings treated below 700 °C, the transmittance is almost independent of the pyrolytic temperature, owing to the amorphous matrix with a rather homogeneous structure, while the composite coatings treated over 700 °C show a slight decline in transmittance with increasing temperature. An increase in annealing temperature leads to a rougher coating structure, which probably results in an increase in light scattering intensity, hence causing a decrease in transparency.

Fig. 8(c) displays the surface hardness and elastic modulus values of the composite coatings as a function of the pyrolytic temperature. With an increase in temperature, an obvious enhancement in the surface hardness and elastic modulus values of the coatings is observed. The surface hardness and elastic modulus of coatings treated at 400 °C are 2.49 GPa and 30.42 GPa, while the hardness and elastic modulus of coatings treated at 900 °C increase up to 9.45 GPa and 83.66 GPa. These are at the same level as SiO_2_–TiO_2_ multilayers prepared *via* a reactive radio frequency (RF) magnetron sputtering method, a vapor deposition method producing mechanically robust film. For example, the hardness and modulus of a SiO_2_–TiO_2_ multilayer coating reported by M. Mazur *et al.* are 8.3 GPa and 73.0 GPa, which are considered higher than glass,^45^ while the hardness and modulus of a SiO_2_–TiO_2_ coating reported by I. Pana *et al.* are 7.18 GPa and 88.31 GPa, respectively.^46^ These results demonstrate that the SiO_2_/TiO_2_ film is dense and mechanically robust. The enhancement in the hardness and elastic modulus with an increase in pyrolytic temperature is correlated with the denser structure of the coating obtained at higher temperatures.

The hydrophilic properties of the coatings were evaluated by examining their water contact angles (Fig. 8(d)). It can be seen that the water contact angles of the coatings obviously decrease with an increase in pyrolytic temperature, and that of the sample annealed at 900 °C is only 38°. The reduction in the contact angle with an increase in treatment temperature can be explained as follows: on the one hand, the higher roughness of the coatings obtained at higher temperatures leads to an enhancement in the contact area between H_2_O and the surfaces of the coatings; on the other hand, according to the XPS analysis, the OH^−^ content is enhanced with an increase in pyrolytic temperature. A higher OH^−^ content causes an enhancement of van der Waals forces and hydrogen bonding interactions between H_2_O and OH^−^.^40^

## 4. Conclusions

Dense, smooth SiO_2_/TiO_2_ coatings with a uniform distribution of Ti element have been successfully prepared with a modified sol–gel method *via* the hydrolysis and subsequent pyrolysis of a single precursor source of TBT-modified PHPS under air flow. The pyrolytic temperature has a significant effect on the compositions and microstructures of the ceramic coatings, and thereby greatly affects their optical, mechanical, and hydrophilic properties. A high Si–O–Ti bond content has been achieved with this single precursor source for SiO_2_ and TiO_2_, which thus ensures homogeneity in the final coating. A dense amorphous silica matrix from PHPS plays an important role in dispersing the Ti moieties and gives the obtained coatings a smooth surface. Therefore, the coatings prepared at various temperatures have a high transparency of over 90% in the visible region, and a refractive index in the range of 1.62 to 1.70 at a wavelength of 630 nm. With an increase in the pyrolytic temperature from 400 to 900 °C, the surface hardness of the coatings drastically increased from 2.49 to 9.45 GPa, and the elastic modulus of the coatings increased from 30.42 to 83.66 GPa. In summary, this work has demonstrated an effective strategy to prepare dense, smooth, homogeneous SiO_2_/TiO_2_ coatings, which have high potential for industrial applications.

## Conflicts of interest

There are no conflicts to declare.

## Supplementary Material
